# Microfabrication of Micropore Array for Cell Separation and Cell Assay

**DOI:** 10.3390/mi9120620

**Published:** 2018-11-24

**Authors:** Yaoping Liu, Han Xu, Lingqian Zhang, Wei Wang

**Affiliations:** 1 Institute of Microelectronics, Peking University, Beijing 100871, China; yaopingliu@pku.edu.cn (Y.L.); h.xu@pku.edu.cn (H.X.); zlqpku@gmail.com (L.Z.); 2 R&D Center of Healthcare Electronics, Institute of Microelectronics, Chinese Academy of Sciences, Beijing 100029, China; 3 National Key Laboratory of Science and Technology on Micro/Nano Fabrication, Beijing 100871, China

**Keywords:** micropore array, Parylene-C, molding, cell separation, cell assay

## Abstract

Micropore arrays have attracted a substantial amount of attention due to their strong capability to separate specific cell types, such as rare tumor cells, from a heterogeneous sample and to perform cell assays on a single cell level. Micropore array filtration has been widely used in rare cell type separation because of its potential for a high sample throughput, which is a key parameter for practical clinical applications. However, most of the present micropore arrays suffer from a low throughput, resulting from a low porosity. Therefore, a robust microfabrication process for high-porosity micropore arrays is urgently demanded. This study investigated four microfabrication processes for micropore array preparation in parallel. The results revealed that the Parylene-C molding technique with a silicon micropillar array as the template is the optimized strategy for the robust preparation of a large-area and high-porosity micropore array, along with a high size controllability. The Parylene-C molding technique is compatible with the traditional micromechanical system (MEMS) process and ready for scale-up manufacture. The prepared Parylene-C micropore array is promising for various applications, such as rare tumor cell separation and cell assays in liquid biopsy for cancer precision medicine.

## 1. Introduction

Micropore arrays have attracted lots of attention due to their capability in cell operations at the single cell level, especially rare tumor cell separation and cell assays from a large volume of clinical samples in the liquid biopsy. The competitiveness of micropore array-based filtration among the developed techniques for liquid biopsy is its promising potential to realize a high throughput at mL/min, along with a high recovery rate [1,2,3,4]. To fulfill the above challenges, the micropore array needs to possess the following properties: (1) a uniform size, geometry, and density of pores to ensure a high separation precision and a high recovery rate; (2) a large area and a high porosity, i.e., a small sized supporting structure (edge-to-edge space) between the adjacent pores to realize a high filtration throughput for the efficient operation of large-volume clinical samples; and (3) a small edge-to-edge space (high porosity) to achieve a high purity of target cells via eliminating the non-specific adhesion of non-target cells to facilitate the downstream analysis, such as gene sequencing and drug screening.

A symmetric review of the reported micropore arrays is summarized in Table 1. The earliest reported micropore array is the polymer filtration membranes prepared via a track-etched method, which can be traced back to the 1960s [5,6], and has been widely utilized in biological studies and clinical practice for cell enrichment [7,8]. For the track-etched micropore array, it is easy to realize a large area; however, the size and geometry are uncontrollable (with fusion of two or more pores), and the porosity is very low (less than 1%), resulting from the random placement of pores with a relatively low density. During the last decade, several strategies via microfabrication techniques for micropore arrays have been developed, with a precisely controlled size, geometry, and density of pores [9,10,11,12,13,14,15,16,17,18,19,20,21,22,23]. Si micropore arrays with an area of 0.64 cm^2^ and porosity of 10% were produced via deep reactive iron etching (DRIE) and KOH etching approaches by Wit et al. [9]. SU-8 micropore arrays with an area of a 9-mm circle and a porosity of <12.5% were fabricated by Adams et al. [10,11,12]. A tapered slit array of SU-8 with a porosity <11% was realized by Kang et al. [13]. A poly(ethylene terephthalate) (PET) microcavity array [14] and nickel (Ni) [15] micropore array were prepared via laser drilling and photolithography-based electroforming, respectively, by Hosokawa et al., with a porosity of <2.25%, although the area was not difficult to extend to >1 cm^2^. A mechanically strong polyethylene (glycol) diacrylate (PEGDA) filter containing conical hole arrays via ultraviolet (UV)-assisted molding with an area of a 6−9 mm circle and a porosity of <5.88% was fabricated by Tang et al. [16]. Fan et al. utilized a sandwich molding technique (modified soft lithography) for the preparation of a polydimethylsiloxane (PDMS) micropore-arrayed membrane from a microfabricated silicon micropillar-arrayed master. This approach was cost-effective and could potentially be used for large-area fabrication (2.25 cm^2^). However, the space between the adjacent pores in the produced membrane exceeded 14 μm, and its porosity was still less than 20% [17]. Several types of Parylene C filtration membrane of microspring structures [18], rectangular-pore arrays (porosity of 18%) [19], and circular-pore arrays (area of 1 cm^2^ and porosity of <5.6%) [20] were obtained via photolithography-based micropatterning and oxygen plasma etching. Three-dimensional (3D) double-layered Parylene C micropore arrays (area of 1 cm^2^ and porosity of <6.96%) [21] and 3D palladium micropocket arrays [22] (area of 1 cm^2^ and porosity of <5.02%) were also reported.

From the above, the uniformity in size and geometry of the previously reported micropore arrays have already presented a good controllability, benefiting from the microfabrication techniques. Additionally, a relatively large area was achievable for some of the mentioned approaches [8,13,14,15,17,20,21,22]. However, the low porosity, resulting from a relatively large edge-to-edge space between the adjacent pores, was still a serious challenge for all the reported micropore arrays; while it is critical for a high filtration throughput, a critical index in the operation of large-volume clinical samples. The elevation of porosity was restricted by the mechanical strength and the process difficulty, considering that the high porosity of micropore arrays may cause a large deformation at a high filtration throughput, resulting in a decrease of the size separation accuracy. In short, a microfabrication technique for uniformly packed micropore arrays of a large area and a high porosity is urgently required.

**Figure 1:**
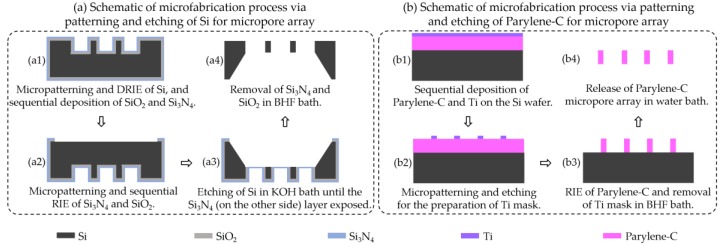
Schematic of microfabrication process via micropatterning and etching of Si (**a**) and Parylene-C (**b**) for micropore arrays preparation.

**Figure 2:**
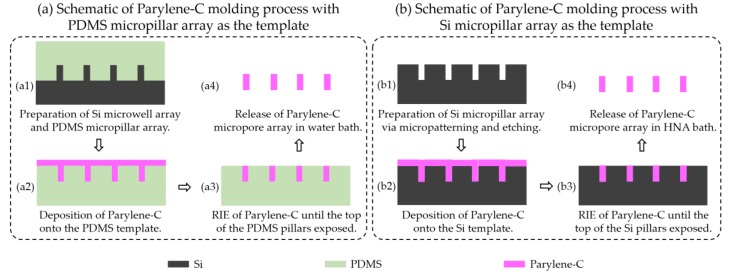
Schematic of the Parylene-C molding process with a polydimethylsiloxane (PDMS) (**a**) and Si (**b**) micropillar array as templates for micropore array preparation.

**Figure 3:**
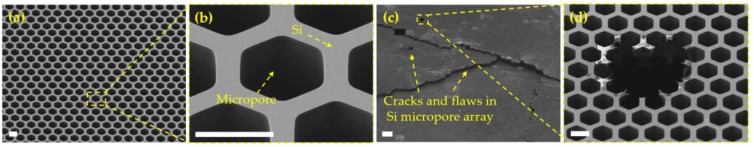
The prepared silicon micropore arrays via micropatterning and etching. (**a**) The well-prepared silicon micropore array; (**b**) the amplification view of the squared area in (**a**); (**c**) defects (cracks and flaws) in silicon micropillar arrays due to the poor mechanical strength; (**d**) the amplification view of the squared area in (**c**). The scale bars are 10 μm (**a**,**b**,**d**) and 100 μm in (**c**).

**Figure 4:**
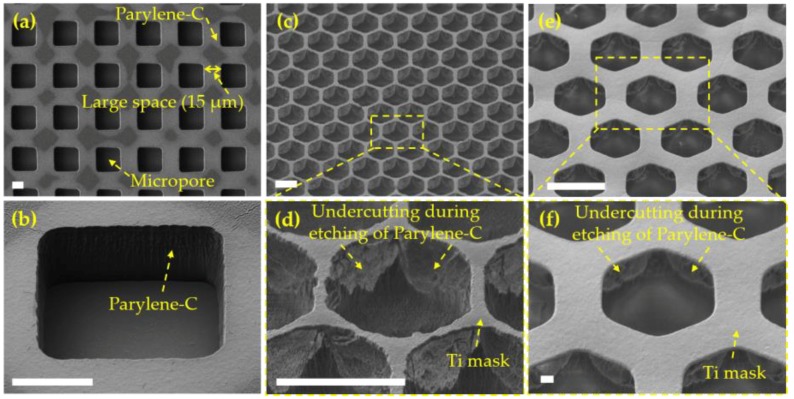
The prepared Parylene-C micropore arrays via micropatterning and etching. (**a**) The well-prepared Parylene-C micropore array with a large space; (**b**) the oblique view of a single micropore in (**a**); (**c**) the SEM images of Parylene-C and Ti (prepared via BHF etching) after RIE; (**d**) the amplification view of the squared area in (**c**); (**e**) the SEM images of Parylene-C and Ti (prepared via RIE) after RIE; (**f**) the amplification view of the squared area in (**e**). The scale bars are 10 μm.

**Figure 5:**
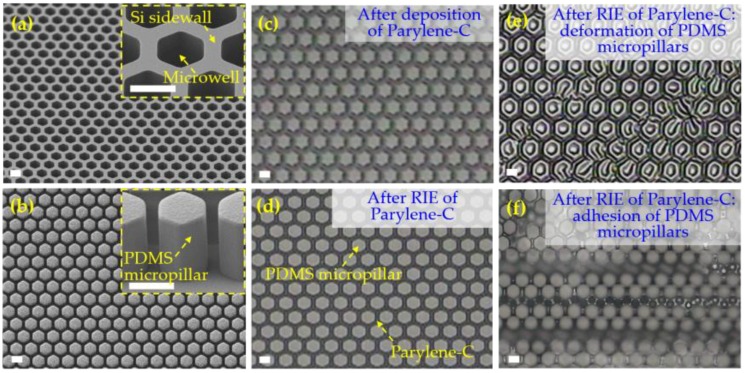
Fabrication results of the Parylene-C molding process with the PDMS template. (**a**) Silicon microwell array prepared via photolithography-based micropatterning and DRIE; (**b**) micropillar array of PDMS prepared via soft lithography with a silicon microwell array as the master; (**c**) PDMS micropillar array after Parylene-C deposition on the top surface; (**d**) PDMS micropillar array with Parylene-C left in the edge-to-edge spacing areas after RIE removal of Parylene-C on the top surfaces; (**e**) deformed PDMS pillars after RIE of Parylene-C; (**f**) adhered PDMS pillars after RIE of Parylene-C. The scale bars are 10 μm.

**Figure 6:**
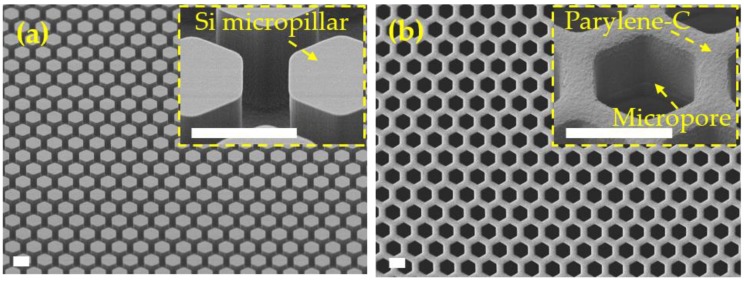
Fabrication results of the Parylene C molding process with a silicon template. (**a**) Si micropillars prepared via photolithography micropatterning and DRIE; (**b**) the prepared Parylene-C micropore array. The scale bars are 10 μm.

**Figure 7:**
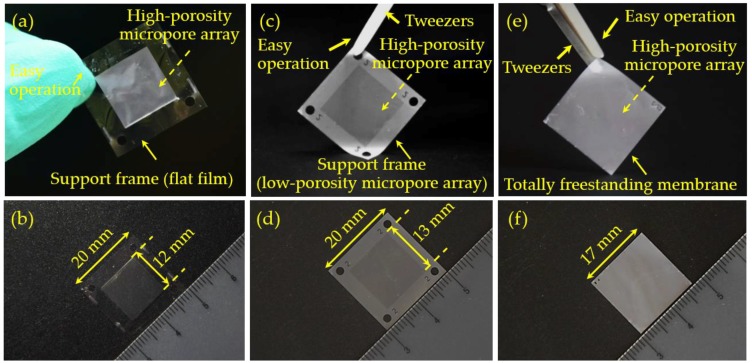
Photos of micropore-arrayed membranes prepared via the Parylene-C molding process with an Si micropillar array as the template. (**a**,**b**) 1st generation: with 4 mm wide flat film as a support frame in the surrounding area and effective filtration (high-porosity micropore array) in the central area; (**c**,**d**) 2nd generation: with a low-porosity micropore array (4 μm pore diameter and 4 μm edge-to-edge space) as a support frame in the surrounding area and effective filtration (high-porosity micropore array) in the central area; (**e**,**f**) 3rd generation: totally freestanding membrane with a high-porosity micropore array in the whole area.

In this study, four different approaches for preparation of the demanding large-scale and high-porosity micropore arrays were investigated in parallel for comparison. They include the micropatterning and etching of silicon (Figure 1a), micropatterning and etching of Parylene-C (Figure 1b), Parylene-C molding with a PDMS micropillar array as the template (Figure 2a), and the Parylene-C molding technique with a silicon micropillar array as the template (Figure 2b). The fabricated micropore arrays via the above four processes were displayed in Figure 3, Figure 4, Figure 5, Figure 6, respectively. Furthermore, three different layout designs of micropore-arrayed membranes in the Parylene-C molding technique with a silicon template were tried, in order to acquire the optimal version (Figure 7).

## 2. Materials and Methods

### 2.1. Micropatterning and Etching of Silicon for Micropore Array Preparation

The micropatterning and etching process for silicon micropore arrays is schematically shown in Figure 1a. First, the lithography-based patterning and DRIE of a 20 μm depth on the top surface of a double-polished silicon wafer were performed, followed by the sequential depositions of SiO_2_ (thickness at 1000 Å) and Si_3_N_4_ (thickness at 1000 Å) via low pressure chemical vapor deposition (LPCVD), as shown in Figure 1a1. Then, photolithography-based micropatterning and reactive ion etching (RIE) of Si_3_N_4_ and SiO_2_ were performed on the bottom surface of a silicon wafer (Figure 1a2). Next, a KOH bath was used to etch the silicon from the bottom surface until the SiO_2_ layer was removed, and the Si_3_N_4_ (on the other side) layer was exposed, sequentially (Figure 1a3). Finally, silicon micropore arrays of a 20 μm thickness were obtained after the removal of SiO_2_ and Si_3_N_4_ in a buffered hydrofluoric acid (BHF) bath (Figure 1a4).

### 2.2. Micropatterning and Etching of Parylene-C for Micropore Array Preparation

The micropatterning and etching process of Parylene-C for micropore array preparation is schematically shown in Figure 1b. First, Parylene-C of a 10 μm thickness and titanium (Ti) of a 3000 Å thickness were sequentially deposited on a single-polished silicon wafer (Figure 1b1). Then, photolithography and etching (with RIE and BHF, in parallel for comparison) were performed to prepare the Ti mask for the subsequent etching of Parylene-C (Figure 1b2). Next, RIE of oxygen plasma was used to etch Parylene-C until the surface of silicon wafer was exposed, followed by the use of a BHF bath for the removal of residual Ti (Figure 1b3). Finally, the release of Parylene-C micropore arrays from the silicon wafer was realized via sonication in the water bath (Figure 1b4).

### 2.3. Parylene-C Molding with PDMS Micropillar Array as the Template for Micropore Array Preparation

The schematic of the Parylene-C molding process with a PDMS micropillar array as the template is schematically shown in Figure 2a. The PDMS micropillar arrays were prepared via the widely used soft lithography technique. First, silicon microwell arrays (depth at 10 μm, and space at 4 μm) were prepared via photolithography-based micropatterning and DRIE on a single-polished wafer, followed by soft lithography to prepare the PDMS micropillar array (Figure 2a1). Then, Parylene-C of a 3 μm thickness was deposited onto the PDMS substrate with a micropillar array with a commercial deposition machine (PDS 2010, SCS, Indianapolis, IN, USA) (Figure 2a2). Next, oxygen plasma etching was used to remove Parylene-C until the top of the silicon micropillars was exposed (Figure 2a3). Finally, sonication in a water bath was performed to release the Parylene-C micropore arrays from the PDMS template (Figure 2a4).

### 2.4. Parylene-C Molding with Silicon Micropillar Array as the Template for Micropore Array Preparation

The schematic of the Parylene-C molding process with a silicon micropillar array as the template is shown in Figure 2b, and this process was also discussed in our previous work [23,24]. First, photolithography-based micropatterning and DRIE were used to fabricate the micropillar array (height at 10 μm, and space at 4 μm) on a single-polished silicon wafer (Figure 2b1). Then, Parylene-C of a 3 μm thickness was deposited onto the silicon template with micropillar arrays (Figure 2b2). Next, oxygen plasma etching was used to etch Parylene-C off until the top of the silicon micropillars was exposed (Figure 2b3). Finally, the release of Parylene-C micropore arrays from the silicon template was realized in the HNA (HF:HNO_3_:HAc = 5:7:11, *v*/*v*) bath (Figure 2b4).

For the Parylene-C molding process with a silicon template, three different layout designs of micropore-arrayed membranes were tried, in order to acquire the optimal version for the ease of operation and application performance maximization.

## 3. Results and Discussion

### 3.1. Micropore Arrays Obtained from Micropatterning and Etching of Silicon

The fabricated micropore arrays via the micropatterning and etching of silicon are displayed in Figure 3. The microfabrication process of silicon in a microelectromechanical system (MEMS) is well-developed, and a high size controllability of microstructures could be maturely realized. The uniformly close-packed silicon micropore array with edge-to-edge space <4 μm (porosity > 40%) was fabricated, as shown in Figure 3a,b. However, the fragility of silicon led to the generation of cracks, and the unavoidable defects in the Si_3_N_4_ layer caused the formation of flaws (Figure 3c,d with three cracks and five flaws in an area of 5.24 mm^2^), thus resulting in a low yield for large-area micropore array microfabrication. The frequently appearing defects result in a questionable mechanical strength of large-area and high-porosity silicon micropore arrays, which will degrade operation ease and cell separation efficiency in practical applications. Besides, the cost of a large-area silicon micropore array is too high, limited by the expensive DRIE, and is still not ready for manufacturing for wide applications. Therefore, a cost-effective alternative microfabrication process for large-area and high-porosity micropore array preparation needs further improvement and development.

### 3.2. Micropore Arrays Obtained from Micropatterning and Etching of Parylene-C

Parylene-C is a popular polymer material for MEMS devices owing to its good biocompatibility and compatibility with the conventional microfabrication processes. Previously, Parylene-C micropore arrays have been reported [18,19,20,21] for cell separation. The reported Parylene-C micropore arrays with a large edge-to-edge space (10/12 μm [20], 11/12 μm [21], low porosity) were fabricated via micropatterning and etching with the photoresist or metal (aluminum [25,26] or titanium [27]) as the etching mask. Our previous study investigated SF_6_ optimized oxygen plasma etching of Parylene-C [27] with micropatterned Ti acting as the etching mask, and this process was further utilized to try the fabrication of high-porosity Parylene-C micropore arrays in this study. The fabrication results are shown in Figure 4. As shown in Figure 4a,b, the Parylene-C micropore array of a large edge-to-edge space (>15 μm) could be obtained. However, the fabrication of a high-porosity Parylene-C micropore array with a small (4 μm) edge-to-edge space failed due to the undercutting during the etching process (Figure 4c−f), resulting from the limited anisotropic etching capability of Parylene-C and thus the difficulty in obtaining a high-aspect-ratio microstructure. The undercutting of Parylene-C in a small sized area was inevitable, even though the quality of the Ti mask was improved via RIE (Figure 4e,f), compared to that prepared via BHF etching (Figure 4c,d). From the above, a process for the microfabrication of structures with a high aspect-ratio is required to obtain high-porosity Parylene-C micropore arrays.

### 3.3. Micropore Arrays Obtained from Parylene-C Molding with PDMS Template

To fabricate the high-aspect-ratio Parylene-C microstructures, a Parylene C molding process was developed by Suzuki et al. [28] and Kuo et al. [29] to prepare the suspended microsprings and microbeams with a size of >10 μm. Our previous study used the molding process for a large-area and high-porosity micropore array [23,24]. The reported Parylene-C molding fabrications used the expensive silicon microstructure as the template. Considering the fabrication cost, the Parylene-C molding process with an economic PDMS microstructure as the template was investigated in this study. The fabrication results are shown in Figure 5. A large-area (20 × 20 mm) PDMS micropillar array with space at 4 μm could be well-prepared (Figure 5b) via the widely used soft lithography process with the silicon microwell array (Figure 5a) as the master. The cost of the PDMS micropillar arrayed template is very low owing to the repeatability uses of the silicon microwell arrayed master. After the deposition (Figure 5c) and RIE removal (Figure 5d) of Parylene-C, the micropore array was expected to be released via a sonication performance in the water bath. However, in fact, the release failed, even after 24 h continuous sonication, which may be attributed to the fact that the Parylene-C molecules were imbedded in the porous molecular network of the PDMS matrix during deposition and thus displayed a very tight adhesion to the PDMS micropillars. Besides, a serious heating effect existed in long-term oxygen plasma etching, which caused the deformation (Figure 5e) and even adhesion (Figure 5f) of PDMS micropillars under the vacuum pumping. Finally, the Parylene-C molding process with the PDMS micropillar array as the template failed to produce the required micropore arrays, and improvement of the process is still in demand.

### 3.4. Micropore Arrays Obtained from Parylene-C Molding with Silicon Template

After the poor capabilities or even failures in the preparation of large-area and high-porosity micropore arrays, the Parylene C molding process with a silicon micropillar array as the template was used as the final ability to fabricate the required micropore arrays. The fabrication results are shown in Figure 6. A large-area (>17 × 17 mm) micropore array with edge-to-edge space <4 μm (porosity >40%) was successfully achieved. The smallest size tried could go down to 1.39 ± 0.07 μm based on the conventional UV lithography. A steep profile of the sidewall was obtained (the insert of Figure 6b), which fulfills a good uniformity of pore size, and thus a high precision/efficiency in cell separation and cell assays in practical applications. The Parylene-C micropore-arrayed membrane presents a high mechanical strength and easy operation, either with tweezers or manually (Figure 7). Besides the preliminary version of the Parylene-C micropore-arrayed membranes in our previous reports [24,25], different layout designs were investigated and compared from the concerns of mechanical strength, easy operation, and filtration performance, as shown in Figure 7. The 3rd third generation of a totally freestanding membrane with a 100% effective filtration area is the optimal design, which could process 10 mL (the largest volume we ever tried) undiluted whole blood without clogging, and more investigations of various clinical samples are ongoing.

Thanks to the strong robustness and high yield of this process (Table 2), it is ready for a scale-up manufacture in a foundry, which would lower the fabrication cost. The yield of this process could be as high as 85%, representing the ratio of the number of membranes without defects to the total number of membranes (both without and with defects) after verification under a microscope. Therefore, we consider that with the improved and optimized batch fabrication, this technology can be very cost effective, which makes it suitable to develop, or to be integrated into, a device for cell separation and cell assays in the clinics.

## 4. Conclusions

In this study, four different microfabrications of micropore arrays for cell separation and cell assays were investigated and compared in terms of the performances in size precision and controllability, robustness, and yield (Table 2). The Parylene-C molding process with a microfabricated silicon micropillar array as the template is the optimized one to prepare the widely required large-area (>17 × 17 mm) and high-porosity (>40%) micropore arrays, along with a high size/geometry controllability (good micropore size uniformity and small edge-to-edge space <4 μm). These advantages make our Parylene-C micropore array attractive in high-efficiency cell separation and cell assays in the liquid biopsy for potential clinical diagnosis and therapy in Precision Medicine. Furthermore, the high yield and strong robustness make this process ready for scale-up manufacture in a foundry, which will be very cost effective and facilitates the broad applications in the fields of basic biomedical study and practical clinics.

## Figures and Tables

**Table 1:** Summary and comparison of the reported micropore arrays.

Ref. No.	Material	Fabrication Strategy	Area	Porosity ^1^	Edge-to-Edge Space
8	Polycarbonate	Track-etching technique	1 cm^2^	N/A	N/A (Randomly distributed)
9	Silicon	Photolithography-based micropatterning, and DRIE and KOH etching	0.64 cm^2^	10%	9 μm
10−12	SU-8	Photolithography-based micropatterning	0.64 cm^2^	<12.5%	13 μm
13	SU-8	Photolithography-based micropatterning	1 cm^2^	6.2%/11%	N/A
14	PET	Photolithography-based micropatterning and laser drilling	4 cm^2^	0.008%	58 μm
15	Ni	Photolithography-based micropatterning and electroforming	1 cm^2^	0.64%	51 μm
16	PEGDA	Micropatterning and UV-assisted molding	0.81 cm^2^	3.25−5.88%	22−24.5 μm
17	PDMS	Modified soft lithography	2.25 cm^2^	20%	14.2−18.1 μm
18	Parylene-C	Photolithography-based micropatterning and oxygen plasma etching	0.5 cm^2^	N/A	N/A
19	Parylene-C		0.36 cm^2^	18%	N/A
20	Parylene-C		1 cm^2^	<5.6%	10/12 μm
21	Parylene-C		1 cm^2^	<6.96%	11/12 μm
22	Palladium	Photolithography-based micropatterning and electroforming	1 cm^2^	5.02%	4/26 μm

^1^ The definition of porosity is the ratio of the opening area (i.e., the total area of micropores) to the whole area of the filtration membrane (i.e., the area of micropores plus the area of supporting structures named edge-to-edge space). Some calculations were performed according to the provided parameters in references to extract the values of porosity for comparison.

**Table 2:** Comparison of the four microfabrication processes investigated for micropore arrays.

Process	Size Precision/Controllability	Realization of High Porosity	Process Robustness	Realization of Large Area	Yield
Micropatterning and etching of silicon	High	Yes	Poor	Difficult	Low
Micropatterning and etching of Parylene-C	Low	No	Poor	Achievable	Low
Parylene-C molding with PDMS template	Low	No	Poor	N/A	Low
**Parylene-C molding with silicon template**	**High**	**Yes**	**Strong**	**Easily achievable**	**High**
